# A bird’s-eye view of deep learning in bioimage analysis

**DOI:** 10.1016/j.csbj.2020.08.003

**Published:** 2020-08-07

**Authors:** Erik Meijering

**Affiliations:** School of Computer Science and Engineering & Graduate School of Biomedical Engineering, University of New South Wales, Sydney, Australia

**Keywords:** Deep learning, Artificial neural networks, Bioimage analysis, Microscopy imaging, Computer vision

## Abstract

Deep learning of artificial neural networks has become the de facto standard approach to solving data analysis problems in virtually all fields of science and engineering. Also in biology and medicine, deep learning technologies are fundamentally transforming how we acquire, process, analyze, and interpret data, with potentially far-reaching consequences for healthcare. In this mini-review, we take a bird’s-eye view at the past, present, and future developments of deep learning, starting from science at large, to biomedical imaging, and bioimage analysis in particular.

## 1 Introduction

Ever since the introduction of digital scanning technologies in biological imaging [1], [2], [3], there has been a growing need for powerful computational methods to enable automated quantitative image analysis. Microscopy images potentially contain a wealth of information about the morphological, structural, and dynamical characteristics of tissues, cells, and molecules, which may go unnoticed even to the expert human eye [4], [5], [6]. However, designing computer algorithms to extract this information with high fidelity is a great challenge, as has been well recognized since the mid-1960s, after the first decade of serious attempts [7], [8], [9], and is still true today.

Automated bioimage analysis typically requires executing an intricate series of operations, which may involve image restoration [10], [11], [12] and registration [13], [14], [15], object detection [16], [17], [18], segmentation [17], [19], [20], and tracking [21], [22], [23], as well as downstream image or object classification [24], [25], [26], quantification [27], [28], [29], and visualization [30], [31], [32]. As attested by the just cited reviews and evaluations, a plethora of methods and tools have been developed for this purpose in the first half a century of computational bioimage analysis, based on what may now be considered traditional image processing and computer vision paradigms.

Recently, a major paradigm shift has occurred with the massive adoption of deep learning technologies [33], [34], [35], which are now rapidly replacing traditional data analysis approaches in virtually all fields of science, including bioimage analysis. In a matter of just a few years, the scientific literature on deep learning has grown explosively, not only with research papers describing novel concepts, algorithms, software platforms, and applications, but also with an abundance of reviews and surveys exploring and commenting on the state of the art.

In this mini-review, we take stock and summarize the latest developments and the challenges ahead, starting from science at large, to biomedical imaging, and to bioimage analysis in particular. Rather than providing a technical introduction or an exhaustive review, we briefly discuss major trends in the past, present, and future of deep learning and their implications for bioimage analysis. Along the way, we mainly cite other reviews and surveys for further reading on specific subtopics.

## 2 Deep learning on the rise

**Fig. 1:**
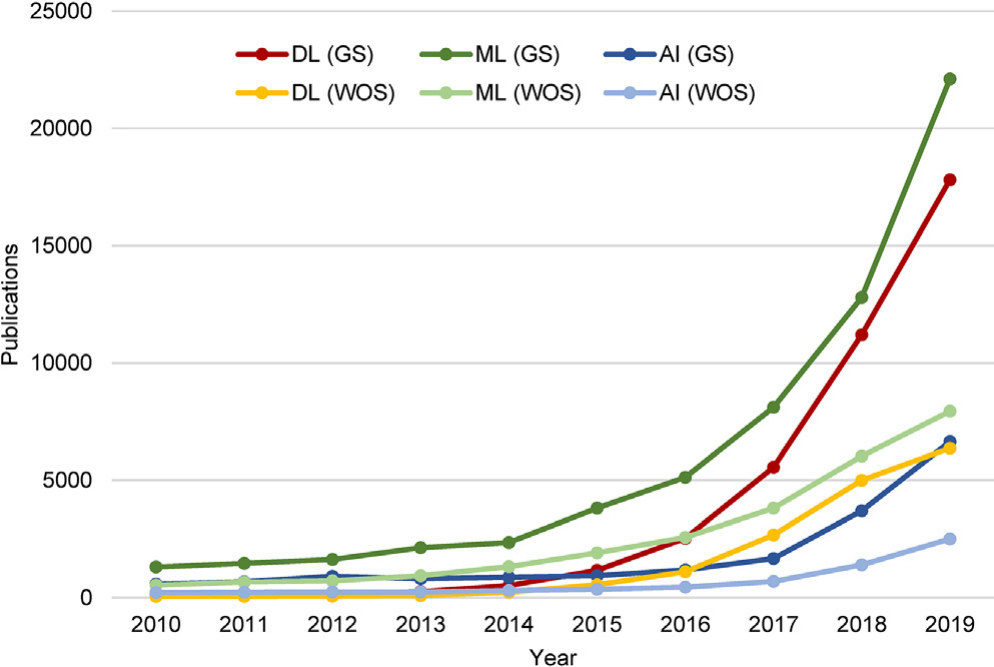
Explosive growth of the scientific literature on deep learning and related topics. The graph shows the number of publications per year in the past decade, having the terms deep learning (DL), machine learning (ML), or artificial intelligence (AI) in the title, according to Google Scholar (GS) and Web of Science (WOS) around the time of submission of this article.

Deep learning popularly refers to the use of artificial neural networks (ANNs) with multiple (ultimately many) layers of elementary computational cells (called “neurons” by analogy with neuronal cells in biological neural networks) to progressively extract higher-level representations of given input data in order to perform data analysis tasks [33], [35], [36]. It is a form of machine learning [34], [37], [38], a major branch of the field of artificial intelligence (AI) [39], [40], [41], which is concerned with the science and engineering of developing machines exhibiting characteristics associated with human intelligence. While deep learning is now taking the world by storm (Fig. 1), its road to success has been long and arduous.

### 2.1 A brief history of deep learning

The idea of using ANNs for data analysis dates back to the dawn of digital computing [42]. In the early 1940s, the first mathematical model of a biological neuron was proposed, providing “a tool for rigorous symbolic treatment of known nets and an easy method of constructing hypothetical nets of required properties” [43]. In order for the model to work, its parameters (weights) had to be set correctly, which initially was done manually. In the late 1950s, the perceptron became the first model capable of learning the weights from examples, illustrating “some of the fundamental properties of intelligent systems” [44]. However, by the end of the 1960s, it was clear that such models have severe limitations [45], and multilayer perceptrons are required for more complex tasks, but it was not obvious how to train them.

After the ensuing first “AI winter”, from the late 1960s throughout the 1970s and some time beyond, interest in ANNs resurged in the mid-1980s with the (re)invention of the back-propagation algorithm [46]. Important advances were made in the 1990s, including the development of recurrent neural networks (RNNs) such as the long short-term memory (LSTM) for modeling data sequences [47], and successful applications of multilayer convolutional neural networks (CNNs) in image analysis [48]. But by the end of the millennium, due to unmet overinflated expectations created by AI-exploiting ventures, and successes in other areas of machine learning, interest in ANNs waned for the second time.

Until the mid-2000s it was generally believed that deep ANNs are very hard to train. This perception started to change when it was shown that a particular type of multilayer ANN called a deep belief network (DBN), where each layer is a restricted Boltzmann machine (RBM), can be efficiently trained by greedy layer-wise unsupervised learning [49]. Soon after, based on the same principle, algorithms for training deep autoencoders (AEs) were proposed [50], as well as other deep architectures [33]. By this time, deep learning began to clearly outperform competing machine learning technologies for various data analysis tasks. This became most evident in the 2012 edition of the ImageNet challenge on image classification, where a CNN called AlexNet won by a large margin [51]. Since then, deep learning has gained ground at an exponential rate, including in the biomedical domain, as covered in later sections of this article.

### 2.2 Driving forces behind deep learning

In recent years, deep learning has been well recognized as a breakthrough technology. So much so that in 2018, the Turing Award, given annually since 1966 by the Association for Computing Machinery (ACM) and generally considered to be the “Nobel Prize of Computing”, was awarded to three highly influential researchers “for conceptual and engineering breakthroughs that have made deep neural networks a critical component of computing”: Yoshua Bengio (University of Montreal), Geoffrey Hinton (University of Toronto & Google), and Yann LeCun (New York University & Facebook) [52].

Apart from groundbreaking research, two other factors have played an important role in the relatively recent rapid rise of deep learning [53]. Both relate directly to the very needs of deep learning algorithms to be successful. The first is the need for large data sets to properly train the likewise large numbers of neural network parameters. Compiling such data sets has been greatly facilitated since the turn of the millennium by the increasing digitization of the world, leading to the present era of “big data”. The second is the need for large computing power to complete the required large numbers of iterations in the training process within reasonable time. More and more advanced computing power has become affordable even for individual researchers in the form of general purpose graphics processing units (GPUs).

Together, these advances have enabled the development of ever deeper neural networks, reaching ever higher accuracies and beating the state of the art in an ever growing number of applications. The widespread usage of deep learning has been further accelerated by the development of open-access software libraries and frameworks [54], [55], [56], [57], greatly facilitating deep neural network (DNN) design and training even for non-computer scientists. Tech giants such as Google, Facebook, Apple, IBM, Intel, Microsoft, Amazon, Baidu, and many others invest heavily in deep learning, capitalizing on its potential and contributing to a world that is increasingly driven by DNNs, and it seems this is only the beginning [58].

### 2.3 Widespread impact of deep learning

The extraordinary power of deep learning in addressing intractable challenges has led to a competitive race for leadership among research groups, universities, companies, and even nations [59]. Every week, new papers appear, not seldom by researchers without a solid background in computer science, commenting on the impact of deep learning in their field, or claiming victory with DNNs in yet another application domain, often simply by exploiting existing software tools and network architectures. The past few years have seen a flood of reviews and surveys on the subject, in virtually all fields of science, often by authors or in journals the seasoned computer scientist had never heard of. Apparently, despite many remaining challenges requiring further research (Section 5), a methodology has emerged that is relatively easy to use and that everyone is eager to own.

By now, deep learning has become the go-to data analysis technology in domains as diverse as agriculture [60], bioinformatics [61], biometrics [62], computational biology [63], consumer analytics [64], cyber security [65], dentistry [66], drug discovery [67], education [68], face recognition [69], gaming [70], health informatics [71], high-energy physics [72], hydrology [73], genomics [74], linguistics [75], mobile multimedia [76], mobile networking [77], multimedia analytics [78], nanotechnology [79], natural language processing [80], precision medicine [81], remote sensing [82], renewable energy forecasting [83], robotics [84], smart manufacturing [85], speech generation [86], surveillance [87], traffic control [88], video coding [89], and countless others [90].

## 3 Deep learning in biomedical imaging

**Fig. 2:**
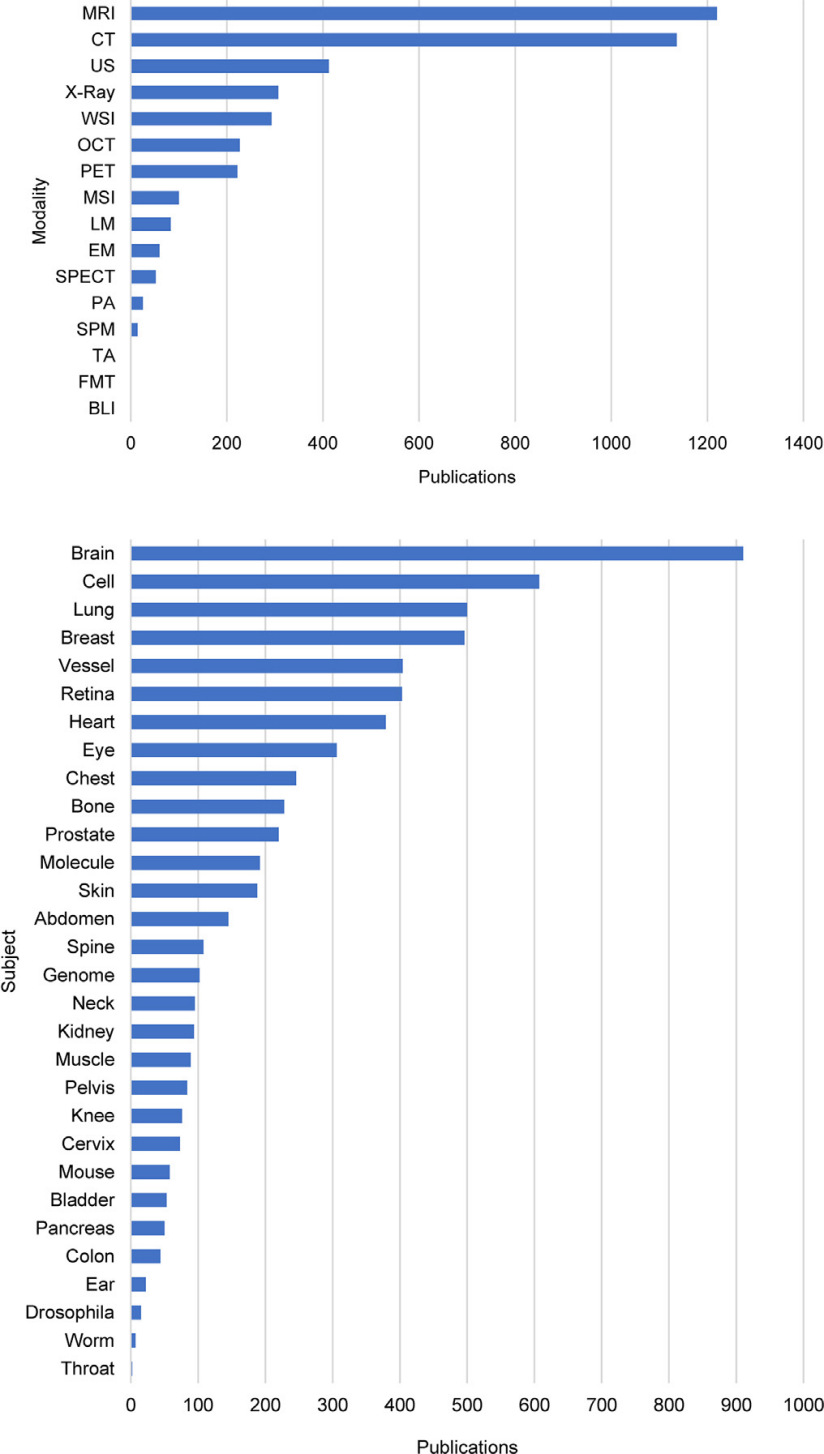
Impact of deep learning on biomedical imaging. The graphs show the number of peer-reviewed journal and selected conference proceedings publications on deep learning in different biomedical application areas, categorized by imaging modality (top, see text for abbreviations) and subject of study (bottom), ranked from most to least popular. Numbers were estimated from the PubMed database of the US National Library of Medicine, National Institutes of Health, around the time of submission of this article, by searching for publications having relevant terms in the title or abstract (Supplementary Data).

A domain we focus on more specifically in this article is biomedical imaging (Fig. 2). Here we take biomedical imaging to be the broad, multidisciplinary field concerned with the acquisition, processing, visualization, and interpretation of structural and functional images of living organisms, whether for clinical or for research purposes. Celebrating a long history of its own [91], including multiple Nobel Prize winning revolutions [92], biomedical imaging has become a cornerstone of modern healthcare and life sciences, to the extent that today “a world without imaging is clearly not imaginable” [93]. For the sake of brevity in this mini-review, we roughly divide the field into medical imaging, pathological imaging, preclinical imaging, and biological imaging in the life sciences, and summarize the impact of deep learning on each.

### 3.1 Deep learning in medical imaging

In clinical practice, the screening, diagnosis, prognosis, and treatment of disease in the human body, all rely increasingly on advanced medical imaging technologies such as X-ray computed tomography (CT), magnetic resonance imaging (MRI), positron emission tomography (PET), single-photon emission computed tomography (SPECT), and ultrasound (US) imaging. Successful application of imaging technologies involves not only high-fidelity image acquisition but also reliable image interpretation [94]. While medical imaging devices have improved substantially in recent decades in terms of sensitivity, efficiency, and image quality, for a long time image interpretation was done primarily by humans. But even experts are known to suffer from subjectivity, variability, and fatigue. These impediments can potentially be overcome by computational methods, and deep learning in particular has emerged as a key enabling technology for this purpose, as attested by many recent overview articles in the field [95], [96], [97], [98], [99], [100], [101], [102].

The impact of deep learning has been reviewed more specifically in a wide range of medical imaging areas, including abdominal imaging [103], atherosclerosis imaging [104], structural and functional brain imaging [105], [106], in-vivo cancer imaging [107], dermatological imaging [108], endoscopy [109], mammography [110], musculoskeletal imaging [111], nuclear imaging [112], ophthalmology [113], pulmonary imaging [114], thoracic imaging [115], as well as in radiotherapy [116], interventional radiology [117], and radiology in general [118], [119], [120]. The massive body of papers on deep learning in virtually all areas of medical imaging has inspired many to write primers [121], [122], [123], guides [124], [125], [126], white papers or roadmaps [127], [128], [129], and other commentaries [130], [131], [132]. There is now growing evidence that deep learning methods can perform on par with, if not better than, radiologists in specific tasks [133], though the latter will continue to play a critical role in integrating such methods in clinical workflows [127].

### 3.2 Deep learning in pathological imaging

Disease diagnosis and prognosis cannot always be performed solely using structural or functional in-vivo medical imaging, but often also require complementary ex-vivo pathological imaging of tissue, cell, and body fluid samples extracted from the body. Perhaps even more than in medical imaging, visual image interpretation in pathology has traditionally been the task of human experts. However, the increasing adoption of digital whole-slide imaging (WSI) into routine clinical practice in recent years has created unprecedented opportunities for computer-aided diagnosis (CAD) in pathology [134], [135], [136], [137]. Here, too, deep learning is being rapidly and widely adopted for this purpose, as reported in many reviews [138], [139], [140], [141], [142], [143], [144], [145].

Pathological imaging plays a prominent role especially in cancer diagnosis and prognosis, and the impact of deep learning has been reviewed in various areas of oncological pathology, including in histopathology [141], cytopathology [146], and hematopathology [147]. Deep learning in pathology has been surveyed more specifically for breast cancer [142], [148], lung cancer [149], [150], tumor pathology in many other forms of cancer [151], and cancer prognosis [152], with many opinion articles commenting on challenges and opportunities [153], [154], [155], [156], [157]. As in medical imaging, there is mounting evidence for the potential of deep learning to provide fast and reliable image analysis at a performance level of a seasoned pathologist, or to serve as a synergistic tool for the latter to improve accuracy and throughput [131].

### 3.3 Deep learning in preclinical imaging

Innovative clinical medical imaging technologies and procedures are usually the fruit of preclinical imaging research with animal models representing humans in studying responses to physiological and environmental changes. Modern small-animal based anatomical, functional, and molecular imaging research involves a wide range of well-established as well as more experimental imaging modalities, including micro versions of clinical scanners (µCT, µMRI, µPET, µSPECT, µUS), optical coherence tomography (OCT), fluorescence molecular tomography (FMT), bioluminescence imaging (BLI), photoacoustic (PA) and thermoacoustic (TA) imaging, multispectral imaging (MSI), and others [93], [158], [159]. The use of deep learning for automated analysis of such imaging data is relatively uncharted territory, but recent studies have reported first applications in translational molecular imaging experiments [160], [161], [162], [163], [164].

### 3.4 Deep learning in biological imaging

Even more fundamental to our understanding of disease processes and the homeostatic mechanisms maintaining life down to the cellular and molecular levels, is biological microscopy imaging, more succinctly also referred to as bioimaging. Revolutionary scientific discoveries and technological innovations in the past decades have spurred the development of a vast array of advanced light microscopy (LM), notably fluorescence microscopy (FM), as well as electron microscopy (EM) and scanning probe microscopy (SPM) imaging modalities that have proven key to much of the progress in modern biological research [165], [166], [167], [168], [169], [170].

Of all biomedical imaging fields, bioimaging arguably faces the biggest challenges in automating visual image interpretation tasks, due to the lack of standard imaging protocols, the high variability of experimental conditions, and the sheer volume of the data produced. Whereas (pre)clinical medical imaging systems typically generate data sets of dozens of megabytes (MB), and digital pathology scanners yield data sets of tens to hundreds of gigabytes (GB), automated microscopes may easily produce on the order of terabytes (TB) of image data in a single experiment [171], [172], [173], [174]. Here, the power of deep learning is increasingly leveraged not only to improve image formation [175], [176], [177], [178], [179], but also subsequent image analysis, discussed next.

## 4 Deep learning for bioimage analysis

**Fig. 3:**
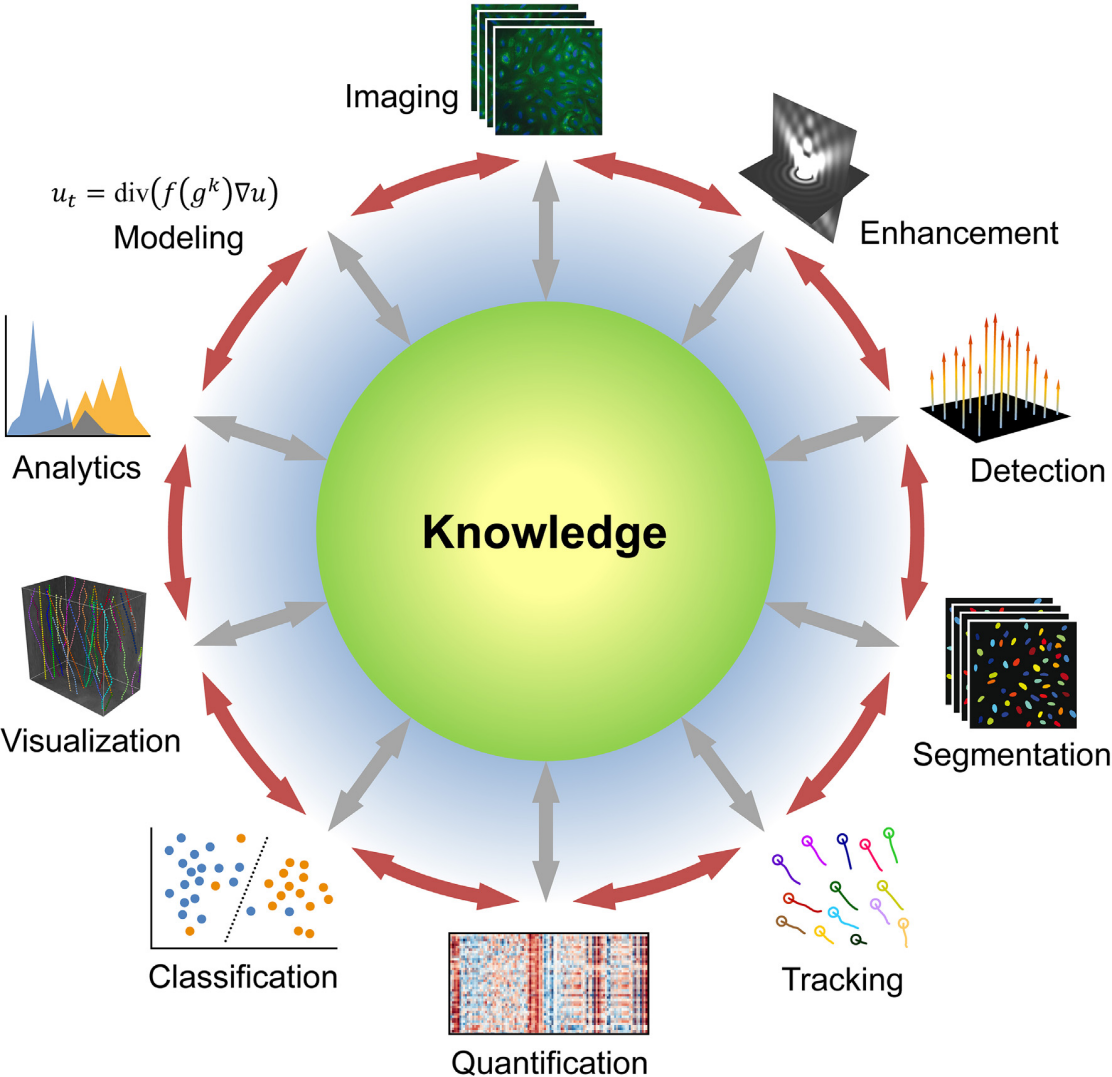
Common tasks in bioimage analysis. The ultimate goal is to gain knowledge of biological processes in health and disease by extracting relevant information from microscopy image or video recordings of these processes. Depending on the specific application, information extraction may involve image enhancement, object detection, image segmentation, object tracking, quantification, and classification, data visualization and analytics, and mathematical or statistical modeling. Deep learning is used increasingly in many of these tasks and we discuss several prominent ones in the main text. The diagram shows a typical order of tasks, with double-headed arrows indicating the possible interrelation and feedback between tasks, as well as the fact that any of them independently may also contribute to knowledge along the way, affecting other tasks. Modified from [6].

First studies using ANNs for bioimage analysis date back to the late 1980s [180], soon after the popularization of the back-propagation algorithm. A review on future trends in microscopy around that time already commented that for complex visual tasks “a good deal of faith is now placed in electronic neural networks” [181]. Indeed, the use of ANNs caught on during the 1990s [182], [183], [184] and 2000s [185], [186], [187], but as in biomedical imaging at large, deep learning began to be massively adopted for bioimage analysis only in recent years [188], [189], [190], [191], [192], [193], [194]. We briefly discuss some of the common tasks in bioimage analysis (Fig. 3) where deep learning has been particularly successful.

### 4.1 Deep learning for image enhancement

**Fig. 4:**
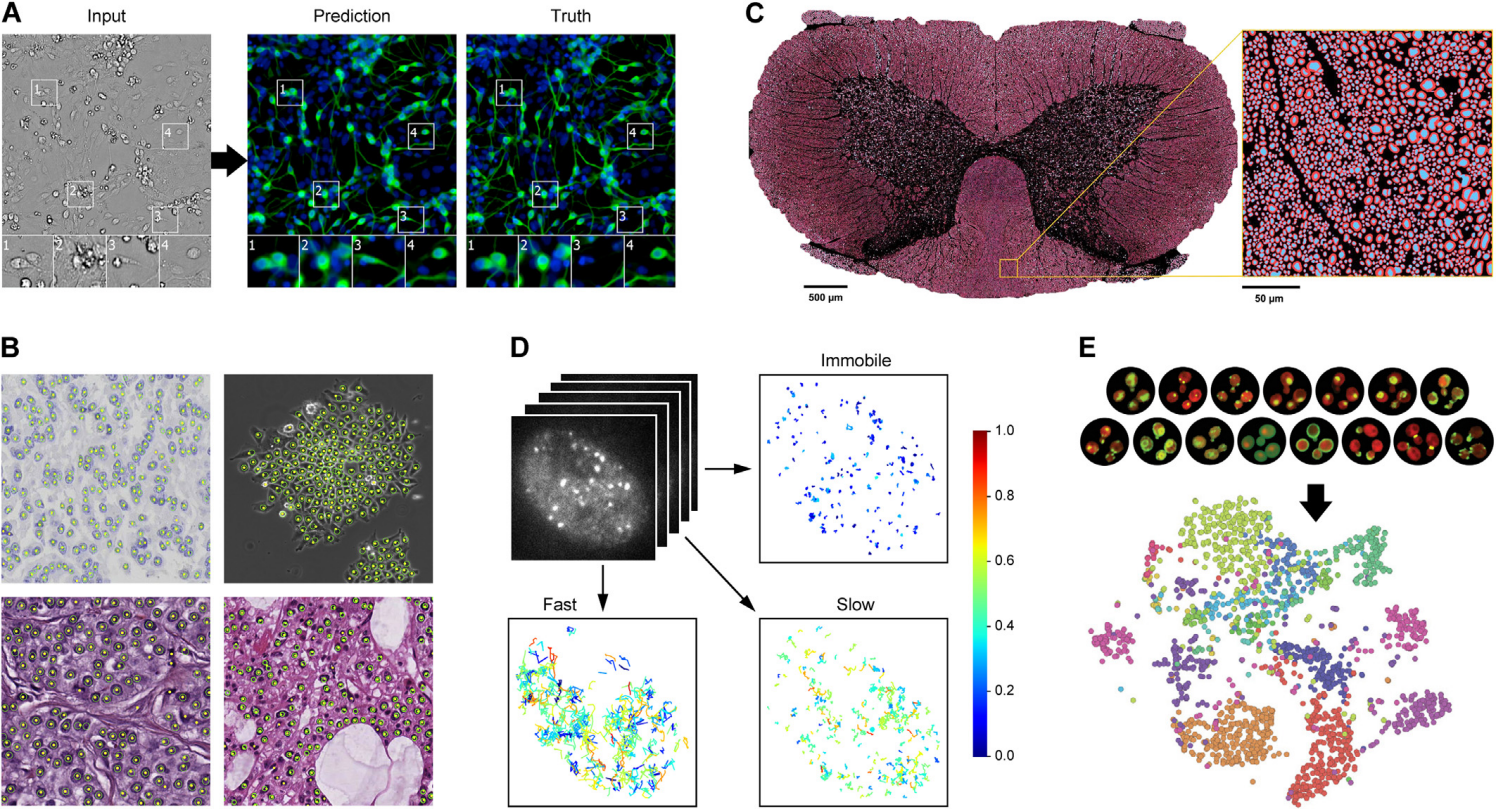
Examples of successful application of deep learning in bioimage analysis. **A**: Prediction of a fluorescence microscopy image (middle) from a bright-field microscopy image (left) compared to the truth (right) [195]. The image shows neurons in a culture of induced pluripotent stem cells differentiated toward the motor neuron lineage but containing other cell types as well. Fluorescent labels are TuJ1 (green) with Hoechst (blue) for the cell nuclei. The predicted image was obtained using a multiscale CNN inspired by U-Net. **B**: Detection of cells in various types of microscopy images [196]: Ki-67 stained bright-field microscopy image of neuroendocrine tumor tissue (top left), phase-contrast microscopy image of HeLa cervical cancer cells (top right), and H&E stained bright-field microscopy images of breast cancer tissue (bottom left) and human bone marrow tissue (bottom right). Detected cells are marked by yellow dots with green circles indicating the ground truth and were obtained using a structured regression model based on a fully residual CNN. **C**: Segmentation of neuronal axons (blue) and myelin sheaths (red) in a full scanning electron microscopy image slice of a rat spinal cord [197]. The segmentation was obtained using a CNN called AxonDeepSeg. **D**: Motion analysis of tracked breast cancer susceptibility gene BRCA2 particles in time-lapse fluorescence microscopy images [198]. Tracks were segmented into tracklets showing consistent motion (no switching between different dynamics states) using an LSTM network. Subsequent moment scaling spectrum (MSS) analysis of the tracklets yielded an estimate of the number of mobility classes (three in this case) and their associated parameters. Color coding indicates the value of the MSS slope per tracklet. **E**: Classification of fluorescence microscopy images (examples at the top) of yeast cells expressing GFP-tagged proteins localizing to 15 subcellular compartments [199]. The classification was done using a CNN called DeepLoc. A visualization (bottom) of the activations of the final convolutional layer of the network in 2D using t-distributed stochastic neighbor embedding (t-SNE) illustrates the power of the model to distinguish the different classes. For more detailed information, see the cited papers, from which the shown examples were adapted with permission (see Acknowledgments section). (For interpretation of the references to color in this figure legend, the reader is referred to the web version of this article.)

Many bioimage analysis tasks are greatly facilitated if the raw microscopy images are first enhanced by removing artifacts and restoring essential information as much as possible. Generally, a high signal-to-noise ratio (SNR) and spatial resolution are beneficial, but may not be achievable in a given experiment due to the required imaging speed and maximum allowable light exposure to avoid damaging the sample. Depending on the type of microscope used and the imaging conditions, different kinds of image enhancement operations may be applied, and deep learning has proven to be a powerful methodology for these. For instance, using well-registered pairs of low-quality and high-quality images, a CNN can be trained to perform denoising and recover resolution [175], [179], [200], [201], [202], [203], [204]. Similarly, trained with pairs of images from different imaging modalities, deep networks can predict fluorescent labels from transmitted-light microscopy images of unlabeled biological samples [179], [195], [205] (Fig. 4A), a technique referred to as cross-modality inference or transformation. Also, generative adversarial networks (GANs) have been shown to enable virtual refocusing of a two-dimensional (2D) fluorescence microscopy image onto a user-defined three-dimensional (3D) surface within a biological sample, correcting for sample drift, tilt, and other aberrations [178].

### 4.2 Deep learning for object detection

Another challenge central to many bioimage analysis tasks is to determine whether certain objects of interest are present in given microscopy images. Object detection often goes hand in hand with object localization and has been the subject of intense research for more than half a century [206]. The problem can be solved by extracting features from local image patches and performing classification on them. Here, too, traditional approaches have made way for deep learning in numerous applications, with two-stage region-proposal CNN-based (R-CNN) and unified you-only-look-once (YOLO) approaches and variants being most popular [207], [208], [209]. In contrast with traditional object detection methods, which have found broad application in bioimage analysis for spotting intracellular particles [16], [18], [210], [211], cell nuclei [17], [26], and cellular events such as mitosis [212], [213], [214], deep learning approaches for these tasks have been explored since only recently. First results are promising [196], [215], [216], [217], [218] (Fig. 4B) but more extensive evaluations are needed to assess their general superiority.

### 4.3 Deep learning for image segmentation

One of the most ubiquitous tasks in bioimage analysis is the partitioning of images into meaningful segments for downstream quantification and statistical evaluation [17], [19], [26]. It is therefore no surprise that the bulk of literature on deep learning in many application areas of computer vision including bioimage analysis has focused on the potential for image segmentation [188], [190], [219], [220], [221], [222]. Similar to object detection, image segmentation can be cast as a classification problem, this time down to the pixel level rather than the object level, which indeed is the approach taken by many deep-learning based methods. In particular, fully convolutional neural networks (FCNs) [223] such as U-Net [224], SegNet [225], DeepLab [226], and variants [227] (Fig. 4C) have become immensely popular for image segmentation. Deep learning methods have also begun to feature prominently in recent international competitions in bioimage analysis, including on segmentation of EM brain images [228], cell nuclei in FM images [229], cells in a variety of time-lapse microscopy images [23], and glandular structures in microscopy images of histological slides [230]. No doubt the future will see more and more deep-learning based methods dominating the charts in such evaluation studies.

### 4.4 Deep learning for object tracking

Characterizing real-life objects requires quantifying not only their spatial properties but also their temporal behavior. As advanced microscopes nowadays enable fully automated acquisition of time-lapse images of living cells and intracellular particles, this calls for robust computational methods capable of not only detecting and segmenting objects, but also tracking them over time in these images. Object tracking is generally considered to be “one of the most challenging computer vision problems” [231] and is a common task also in bioimage analysis [22], [232], [233], [234], [235]. The first international competition of particle tracking methods was held before deep learning broke through, but already led researchers to suggest the development of learning-based tracking methods [21]. Also, the continuing series of cell tracking challenges has seen the increasing use of deep learning methods for the problem [23]. As discussed in recent reviews, much of the work on deep learning for object tracking in bioimage analysis has focused on the spatial aspect of detection and segmentation [190], [193], while the temporal aspect of data association and linking is typically still solved using traditional computer vision methods. First studies have appeared using DNNs to address both [236], [237], as well as for subsequent trajectory analysis [198] (Fig. 4D), but the challenge remains to develop end-to-end deep-learning based cell and particle tracking methods [238].

### 4.5 Deep learning for object classification

The task of identifying images or objects therein as belonging to one of multiple predefined classes is a fundamental problem of computer vision in general [239], [240], [241] and a recurring theme also in bioimage analysis [242], [243], [244]. Traditionally, the problem has been addressed by extracting handcrafted image features and using these together with given class labels to train classifiers such as support vector machines (SVM) or random forests (RF) [25], [245], [246]. But the capacity of deep CNN-based classifiers to learn relevant image features autonomously make them favorable over traditional approaches. Following their great success in the 2012 ImageNet challenge [51], CNN-based approaches have grown in popularity for image classification tasks across the board. In bioimage analysis, they have been shown to achieve expert-level performance in a wide range of cell classification and subcellular pattern recognition tasks [188], [189], [191] (Fig. 4E), although recent evaluations have revealed they do not necessarily outperform traditional approaches [244]. An issue in many studies is the lack of sufficient training data, which may be remedied by leveraging transfer learning [190] or crowd-sourcing strategies [247].

## 5 Summary and outlook

Deep learning has had a long history of discoveries, inventions, expectations, disappointments, rejections, revivals, successes, declines, recoveries, and breakthroughs, but is now widely accepted as the most powerful computing paradigm for big data analysis. The impact of deep learning on our daily lives is already unlike any other technology in the history of computer science, yet it seems we have seen only the proverbial tip of the iceberg. In biomedical imaging, DNNs are beginning to outperform human experts in a growing number of visual interpretation tasks, which is fueling fierce debates among professionals on the future ramifications for the field. Zeroing in on biological imaging, we have reviewed the use of deep learning approaches for common tasks in bioimage analysis, where they are now increasingly favored over traditional computer vision methods. Notwithstanding impressive achievements reported to date, many scientific and engineering challenges remain to further improve deep learning. In closing this mini-review, we touch on several important developments addressing these challenges that are relevant to bioimage analysis (see Table 1 for a quick summary of key research topics with references to reviews and commentaries for further reading).

**Table 1:** Overview of key reviews and commentaries for further reading on big research topics in deep learning (DL).

**Topic**	**References**
Biological DL	Neuro-inspired AI [40] Bio-inspired computer vision [248] Integrating DL and neuroscience [249] Biological vision and ANNs [250]
Optimal DL	User-friendly software platforms [56] Neural architecture search [251] AutoML in biomedical imaging [252]
Economical DL	Semi*/*weakly supervised learning [253] Unsupervised learning strategies [254] Transfer learning strategies [255]
Generalizable DL	Economical DL [256] Open-set recognition [257] Domain adaptation [255]
Multimodal DL	Multimodal learning models [258] Data fusion strategies [259] Omics applications [260]
Efficient DL	Parallelization and distribution [261] Compression and acceleration [262] Biomedical imaging applications [263]
Explainable DL	Interpretable AI approaches [264] Visual analytics tools [265]
Responsible DL	On replacing radiologists [266] On replacing physicians [267] On replacing microscopists [268] Biomedical students on AI [269]

### 5.1 Biological deep learning

Biology has always been a great source of inspiration for technology. Recognizing the unparalleled capacity of the brain in processing information, researchers in computer vision have exploited models of human vision from very early on [248], [270], [271]. Similarly, the idea of developing ANNs for data analysis was born out of research into the workings of biological neural networks (BNNs) [40], [53], [249]. In both cases, however, the ties between computer science and neuroscience have not remained strong, perhaps because “we simply do not have enough information about the brain to use it as a guide” [53]. But as long as human experts continue to be the gold standard in critical vision-based decision-making tasks, it seems there is still much to be gained from renewed interactions between the fields [40], [248], [249], [250]. Bioimage analysis could play a pivotal role here, in a virtuous circle of helping to decipher BNNs at the microscopic level [272], [273], [274] and translating discoveries into improved ANNs for such studies [275], [276], [277].

### 5.2 Optimal deep learning

One of the key strengths of deep learning underlying its great success is that it automates the process of finding optimal feature descriptors given any data analysis task. While this eliminates the cumbersome handcrafting of descriptors, it leaves the user with the responsibility to design the right DNN architecture and tweak its hyperparameters to achieve satisfactory results. In practice, this may still require significant manual effort and yield suboptimal results. Notwithstanding the great arsenal of software toolkits for deep learning available today [54], [55], [56], [57], there is still much room for the development of higher-level, user-friendly platforms that make it easier also for non-experts to adopt and use existing DNNs or to design and deploy their own solutions. The desire to further minimize human intervention in finding optimal solutions has given birth to the field of automated machine learning (AutoML). For deep learning, various neural architecture search (NAS) approaches have been proposed to automate the network engineering process [251], [278], [279]. First successful applications have recently been reported in medical imaging [252], [280], [281], suggesting NAS holds great potential also for bioimage analysis.

### 5.3 Economical deep learning

The most common form of deep learning is supervised learning, which requires input data with corresponding labeled output data. Especially in biomedical imaging applications, the output labels are typically obtained by expert manual annotation of the input data. However, as deep learning methods are notoriously data hungry, preparing a training data set this way can be extremely burdersome. Humans themselves largely learn in an unsupervised fashion, as they “discover the structure of the world by observing it, not by being told the name of every object” [35]. More economical deep learning approaches requiring less human input and*/*or training data are very much needed. Semi-supervised, weakly supervised, and unsupervised learning are important research topics [253], [254], [282] receiving increasing attention also in biomedical imaging [283], [284], [285]. An alternative approach is transfer learning between domains [255], [278], [286] which holds great promise for biomedical imaging as well [287], [288], [289], [290]. Another strategy popular in bioimage analysis is to use high-fidelity simulated data as a surrogate for real data [256], [291], [292], which allows supervised learning with any number of images without requiring manual annotation [293], [294], [295].

### 5.4 Generalizable deep learning

In experimental evaluations of deep-learning methods, visual recognition tasks are typically framed as “closed set” problems, where the possible conditions in the test set are exactly the same as those in the training set. But in many applications, including in biomedical imaging, this is not very realistic. In practice, a more realistic scenario is that “incomplete knowledge of the world is present at training time, and unknown classes can be submitted to an algorithm during testing” [296]. This implies that current claims of superiority of machines over humans must be taken with a grain of salt, and that more generalizable or “open set” approaches to developing and evaluating deep-learning methods are needed. Open-set recognition (OSR) has been studied in the AI literature for some time [257], [296], [297] but has thus far received very little attention in biomedical imaging.

### 5.5 Multimodal deep learning

Nowadays, biomedical studies are hardly ever based on data from one imaging modality alone. Multiple, complementary imaging modalities are often used to obtain a more complete picture of the subject or sample under study. An example in bioimaging is the correlative recording of structural and functional image data, using electron and fluorescence microscopy, respectively [298], [299], [300]. But it does not stop there. Experiments typically also involve collecting genomic, proteomic, metabolomic, or other “omic” information [260], [301], [302], and in clinical studies additional data may come from electronic patient records. To take full advantage of all available information in such studies, powerful multimodal deep learning methods are required. This has been well recognized in various other fields [258], [259], [303] but deserves more attention in bioimaging and calls for an integrative approach to bioimage analysis and bioinformatics.

### 5.6 Efficient deep learning

The ever-growing volume of biomedical data sets and the increasing complexity of DNNs for improved analysis put proportionally higher demands on computing power. Training a deep network to achieve super-human performance, particularly in highly specialized domains such as biomedical imaging, essentially requires super-computing technology. To some extent this is provided by modern multicore GPUs, and more recent tensor processing units (TPUs), which enable single-machine parallelization. But more efficiency is often needed to finish network training within a desired time frame. Codesigning architectural, algorithmic, software, and hardware solutions to allow multi-machine parallelism and scalable distributed deep learning for this purpose is an ongoing engineering challenge [261], [262], [304]. Biomedical imaging at large will greatly benefit from such solutions, as they also facilitate exploiting data from multiple institutes in training DNNs without actually having to share the data, thus mitigating legal or ethical concerns [263], [305], [306].

### 5.7 Explainable deep learning

A major point of criticism for which even the pioneers of deep learning had their early papers rejected by peers in computer science, is that the use of neural networks for any given perceptual task provides “no insight into how to design a vision system” [51]. Even today, many in the community still have a propensity for carefully hand-designed solutions based on a solid understanding of the nature of the task. But the reality is that “methods that require careful hand-engineering by a programmer who understands the domain do not scale as well as methods that replace the programmer with a powerful general-purpose learning procedure” [51]. Nevertheless, the call for more explainability and interpretability of deep learning methods is legitimate and receiving growing attention in many areas of AI research [307], [308], [264] including computer vision [309], [310], [311]. A host of visual analytics tools have been developed to dissect DNNs and uncover what they have actually learned [265], [312], [313]. Such tools have not yet found widespread application in bioimage analysis but could help practitioners better understand the predictions made by network models.

### 5.8 Responsible deep learning

Ultimately, the goal of developing computational image analysis methods for the biomedical domain, from fundamental biological imaging to clinical medical imaging, is to improve the efficacy of healthcare. But in order for biomedical professionals to be willing to transfer their responsibilities to machines, and for those whose health depends on their care to accept such transition, these methods need to be trustworthy enough. In this regard it seems we have not quite reached the tipping point. In the past few years, the question whether or when AI will replace human experts has been pondered in many areas of biomedical imaging [132], [268], [267], [269], [266], [314], [315]. It goes without saying that decision making in biomedicine is more critical and risk-averse than in most other technological domains. Much work remains to take deep learning to the level of transparency, adaptability, creativity, empathy, and responsibility normally required of biomedical specialists. That said, as deep learning methods are already achieving human-competitive performance in specific subtasks and have only just begun showing their considerable potential, DNNs will increasingly play an integral role in biomedical procedures. Historically, “human-machine collaborations have performed better than either one alone” [266], and there are no compelling reasons to believe this will ever change.

## CRediT authorship contribution statement

**Erik Meijering:** Conceptualization, Data curation, Formal analysis, Investigation, Methodology, Resources, Visualization.

## Declaration of Competing Interest

The author declares he has no known competing financial interests or personal relationships that could have appeared to influence the work reported in this paper.
